# Targeting mitophagy for depression amelioration: a novel therapeutic strategy

**DOI:** 10.3389/fnins.2023.1235241

**Published:** 2023-10-06

**Authors:** Wangjun Xu, Weiping Gao, Yukun Guo, Feng Xue, Lulu Di, Shaojie Fang, Linlin Fan, Yangyang He, Yunfeng Zhou, Xinmei Xie, Xiaobin Pang

**Affiliations:** ^1^School of Pharmacy, Henan University, Kaifeng, China; ^2^Henan Key Laboratory of Brain Targeted Bio-nanomedicine, School of Pharmacy, Henan University, Kaifeng, China; ^3^Institutes of Traditional Chinese Medicine, Henan University, Kaifeng, China; ^4^Henan Province Engineering Research Center of High Value Utilization to Natural Medical Resource in Yellow River Basin, School of Pharmacy, Henan University, Kaifeng, China

**Keywords:** depression, mitophagy, mitochondria, drug therapy, regulatory mechanism

## Abstract

Major depressive disorder is a global psychiatric condition characterized by persistent low mood and anhedonia, which seriously jeopardizes the physical and mental well-being of affected individuals. While various hypotheses have been proposed to explicate the etiology of depression, the precise pathogenesis and effective treatment of this disorder remain elusive. Mitochondria, as the primary organelles responsible for cellular energy production, possess the ability to meet the essential energy demands of the brain. Research indicated that the accumulation of damaged mitochondria is associated with the onset of depression. Mitophagy, a type of cellular autophagy, specifically targets and removes excess or damaged mitochondria. Emerging evidence demonstrated that mitophagy dysfunction was involved in the progression of depression, and several pharmacological interventions that stimulating mitophagy exerted excellent antidepressant actions. We provided an overview of updated advancements on the regulatory mechanism of mitophagy and the mitophagy abnormality in depressed patients and animals, as well as in cell models of depression. Meanwhile, various therapeutic strategies to restore mitophagy for depression alleviation were also discussed in this review.

## 1. Introduction

Major depressive disorder (MDD) is a multifactorial psychiatric disorder characterized by persistent feelings of sadness and linked with deleterious effects on cognitive, affective, and physical well-being. Approximately 264 million individuals across the globe, accounting for about 4.5% of the global population, are afflicted with depression (Disease et al., 2018). Furthermore, the global incidence of depression has risen by 28% due to the impact of the COVID-19 pandemic (Collaborators, 2021). The lifetime prevalence of depression fluctuates between 15 and 18%, implying that nearly one in every five persons will undergo an episode at some juncture in their lives (Bromet et al., 2011). Researchers have extensively explored the etiology of depression and put forth diverse hypotheses, encompassing monoamine, neuroendocrine, neurotrophic factors, epigenetic, inflammatory, and hypothalamic–pituitary–adrenal axis hypotheses, etc., (Kim, 2016; Keller et al., 2017; Allen et al., 2018; Zhang G. et al., 2020). However, a definitive theory that comprehensively explicates its pathological mechanism remains elusive. The current first-line antidepressants are predominantly based on the monoamine hypothesis (McCarron et al., 2021). Despite their effectiveness, these medications may take up to 6 weeks to manifest therapeutic effects and frequently give rise to adverse reactions such as headaches, gastrointestinal symptoms, sexual dysfunction, and agitation (Marwaha et al., 2023). Furthermore, approximately one-third to half of depressed patients do not respond to multiple antidepressants (Rush et al., 2009; Cipriani et al., 2018). The two leading diagnostic systems for MDD, namely the Diagnostic and Statistical Manual of Mental Disorders and the International Classification of Diseases, are extensively employed in hospital, outpatient, and community settings (First, 2013; The Lance, 2019). However, these diagnoses should be ascribed only after a single bout of depression lasting for a minimum of 2 weeks, and following the exclusion of other psychiatric diagnoses like anxiety, schizophrenia, and bipolar disorder to ensure that symptoms are exclusively attributed to depression (Malhi and Mann, 2018). Consequently, delving into the etiology of depression from novel perspectives is crucial for guiding clinical diagnosis and facilitate the development of therapeutic interventions.

Mitochondria serve as the “powerhouses” of eukaryotic cells, generating most of the cell’s energy through oxidative phosphorylation in the inner mitochondrial membrane (IMM) to produce adenosine triphosphate (ATP). Moreover, mitochondria assume a pivotal role in upholding intracellular environmental homeostasis through the regulation of reactive oxygen species (ROS), calcium ions (Ca^2+^), and apoptosis (Zorov et al., 2014). Damaged mitochondria can increase the production of mitochondrial ROS (mtROS) (Tripathi et al., 2021), which causes oxidative damage to mitochondrial lipids, DNA, and proteins (Ashrafi and Schwarz, 2013), and also release high levels of Ca^2+^ and cytochrome C into the cytosol, triggering apoptosis (Parsons and Green, 2010). Hence, ensuring the elimination of malfunctioning mitochondria is imperative for the cell’s survival.

**Figure 1:**
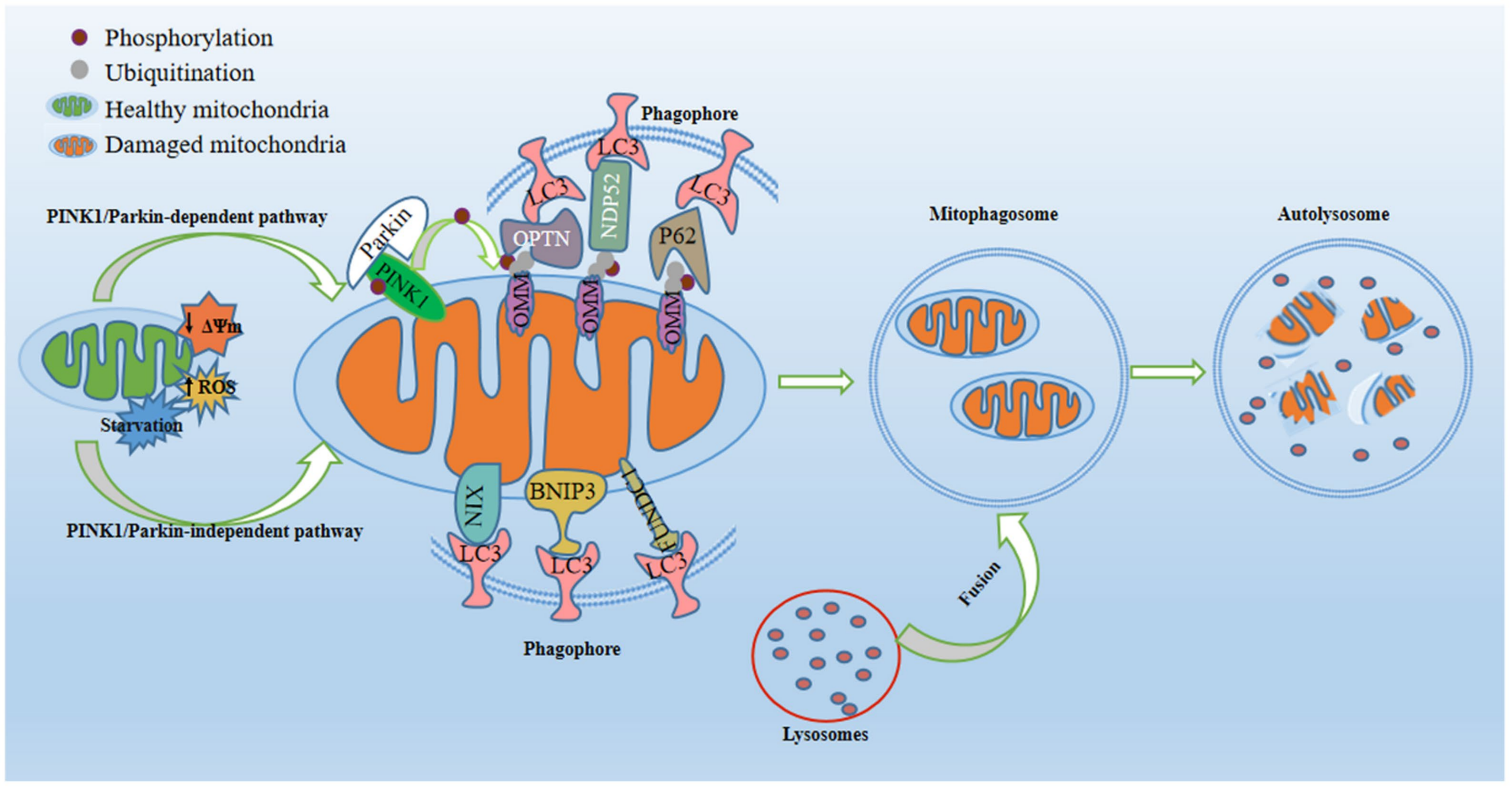
Regulatory mechanisms of mitophagy. Mitophagy, a pivotal process for maintaining mitochondrial quality, is activated in response to mitochondrial damage triggered by conditions such as starvation, diminished mitochondrial membrane potential, or increased reactive oxygen species. In the PINK1/Parkin-dependent pathway, PTEN-induced putative kinase 1 (PINK1) stabilizes on the outer mitochondrial membrane (OMM) and recruits E3-ubiquitin ligase Parkin to the OMM. This prompts the formation of phosphorylated ubiquitin at S65 (p-S65-Ub) on OMM proteins, acting as an “eat-me” signal for damaged mitochondria. Mitophagy receptors (P62, OPTN, and NDP52) recognize and bind to p-S65-Ub, consequently engaging with the phagosome through their LC3-interacting region (LIR) motif, which interacts with LC3 found on the surface of the phagosome. In the PINK1/Parkin-independent pathway, phagophores directly surround mitochondria through OMM receptors containing LIR motifs (NIX/BNIP3L, BNIP3, and FUNDC1) or by detecting exposed cardiolipin on the OMM. Once recruited, the phagophore envelops damaged mitochondria, forming mitophagosomes. Subsequently, fusion between lysosomes and mitophagosomes yields mitolysosomes, culminating in the degradation of dysfunctional mitochondria through acidic hydrolases.

Mitophagy stands as a form of selective autophagy that specifically targets mitochondria, and is widely considered to be the most distinctive type (Galluzzi et al., 2017). Moderate mitophagy effectively eliminates impaired mitochondria, exerting neuroprotective effects, while inadequate or excessive mitophagy may disrupt energy production and impede mitochondria-linked signaling pathways (Yang et al., 2021). Mitochondria depolarize in response to ROS, cellular senescence, nutrient scarcity, and low mitochondrial membrane potential (MMP), thereby triggering mitophagy activation. Defective mitochondria are sequestered by bilayer membrane structures, eventually resulting in the creation of autophagosomes. These specialized vesicles subsequently merge with lysosomes - cellular compartments replete with hydrolytic enzymes - culminating in mitochondrial phagocytosis (Tripathi et al., 2021). Mitophagy ensures the body’s energy metabolism and tissue homeostasis by sequestering damaged mitochondria, balancing mitochondrial mass, and controlling elevated mtROS (Lin et al., 2019), which is mediated by two major pathways, namely PINK1/Parkin-dependent and PINK1/Parkin-independent pathways (Lemasters, 2005; Figure 1). Defects in mitophagy cause the accumulation of dysfunctional mitochondria, precipitating oxidative stress and various pathological conditions. Accumulating evidence substantiates the association between aberrant mitophagy processes and the onset and progression of depression, corroborated by observations of dysfunctional mitophagy in both depressed individuals and mice. Notably, certain antidepressants can alleviate depression-like behaviors in animals by regulating mitophagy. Consequently, rectifying abnormal mitophagy may present an innovative strategy for treating depression.

The objective of this review is to offer a comprehensive overview of the prevailing knowledge concerning the mechanisms of mitophagy and to deliberate on the deviations in mitophagy noted in MDD patients, along with various animal and cellular models of depression. We delineate alterations in biomarkers indicative of mitochondrial dysfunction, autophagy, and mitophagy to underscore the pivotal role played by mitophagy failure in the underlying pathological mechanisms of depression.

## 2. The regulation of mitophagy

### 2.1. The PINK1/Parkin-dependent pathway

The PINK1/Parkin-dependent pathway, governed by PTEN-induced putative kinase 1 (PINK1) and E3-ubiquitin ligase Parkin, has been extensively investigated (Clark et al., 2006). This pathway orchestrates ubiquitin-associated mitophagy, impacting numerous mitochondrial physiological processes, including mitochondrial biogenesis, dynamics, and autophagic machinery (Harper et al., 2018; Pickles et al., 2018). PINK1, a ubiquitin kinase, translocates to the IMM through translocase complexes located on both outer and inner mitochondrial membranes (OMM and IMM), contingent on membrane potential under normal conditions (Jin et al., 2010; Meissner et al., 2011). Subsequently, PINK1 undergoes cleavage by PARL, a resident rhomboid serine protease in IMM (Harper et al., 2018). The resultant N-terminal truncated PINK1 is degraded by the mitochondrial proteasome, and helps in maintaining low levels of PINK1 (Jin et al., 2010; Yamano and Youle, 2013).

The MMP decreases due to mitochondrial damage, which impairs the normal operation of transport enzymes on both IMM and OMM. This impedes PINK1 import and leads to PINK1 accumulation on the OMM. Aggregated PINK1 phosphorylates ubiquitin at S65 (p-S65-Ub) on impaired mitochondria, and consequently drawing cytoplasmic Parkin with a high p-S65-Ub affinity to form ubiquitin chains (Kane et al., 2014). Active Parkin ubiquitinates multiple OMM substrates, yielding more targets for PINK1-driven ubiquitin phosphorylation and fostering further Parkin recruitment (Pickrell and Youle, 2015; Yamano et al., 2016; Malpartida et al., 2021). Mitophagy receptors, like nuclear dot protein 52 kDa (NDP52), sequestosome 1 (SQSTM1, or P62), and optineurin (OPTN), are enlisted where ubiquitin chains have aggregated to a specific level. These mitophagy adaptors feature a ubiquitin-binding domain recognizing ubiquitin chains attached to cargoes, alongside an LC3-interacting region (LIR) enlisting phagophore membranes coated with LC3B, thus initiating mitophagy (Harper et al., 2018).

### 2.2. The PINK1/Parkin-independent pathway

PINK1/Parkin-independent pathways primarily hinge on receptor proteins that directly interact with LC3B and/or gamma-aminobutyric acid receptor-associated protein (GABARAP) through their LIR motifs, precipitating mitochondria elimination. These include like BCL-2 and adenovirus E1B 19-kDa interacting protein 3 (BNIP3), B-cell leukemia/lymphoma 2 (Bcl-2) and adenovirus E1B 19-kDa interacting protein 3-like (NIX), and FUN14 domain-containing 1 (FUNDC1; Doblado et al., 2021).

#### 2.2.1. Bcl-2 family proteins BNIP3 and NIX-mediated mitophagy

Bcl-2 family proteins play a pivotal role in OMM regulation and apoptosis control (Chipuk et al., 2006). Previous studies have shown that these proteins can trigger mitophagy through both Parkin-dependent and Parkin-independent pathways, entailing inhibition of Parkin translocation to depolarized mitochondria and relying on BNIP3 and NIX proteins (Thomas et al., 2011; Hollville et al., 2014). BNIP3 is primarily localized in mitochondria and plays an important role in regulating the fusion of autophagosomes with lysosomes (Ma et al., 2017). NIX (also known as BNIP3L) was cloned from a human placental cDNA library based on its 56% sequence identity to BNIP3 (Matsushima et al., 1998). NIX shares several features with BNIP3, encompassing interaction with BCL2 and BCL-XL, and induction of both apoptosis and autophagy (Chen et al., 1999; Schweers et al., 2007; Novak et al., 2010).

Under hypoxic or starved circumstances, NIX or BNIP3 protein levels surge, orchestrating mitophagy *via* multiple routes. Firstly, these receptors are involved in tethering mitochondria to the autophagosome by directly interacting with LC3 and/or GABARAP on the autophagosome membrane. Secondly, BNIP3 or NIX compete with Beclin-1 to bind BCL-XL. Enhanced NIX expression during erythroid differentiation disrupts existing BCL-XL–Beclin-1 complexes, liberating Beclin-1 and triggering autophagy (Thomas et al., 2011). In addition, BNIP3 can recruit Drp1 and Parkin to mitochondria by binding Parkin, then promoting mitochondrial fission to trigger mitophagy (Lee et al., 2011).

#### 2.2.2. FUNDC1-mediated mitophagy

Previous investigations have identified FUNDC1 as a mitophagy receptor, interacting with LC3B and facilitating its recruitment to mitochondria during mitophagy (Liu et al., 2012). FUNDC1-mediated mitophagy is impeded by phosphorylation at the tyrosine 18 and serine 13 positions under normal physiological conditions. Upon hypoxia stimulation, Src is inactivated and FUNDC1 undergoes dephosphorylation, resulting in increased co-localization and interaction between FUNDC1 and LC3B. This leads to the selective incorporation of mitochondria as cargo into LC3-bound isolation membranes, consequently facilitating mitochondrial removal by LAMP1-positive autolysosomes (Liu et al., 2012; Chen et al., 2014; Lv et al., 2017).

Mitophagy is a complex, multifaceted process characterized by a multitude of molecular, organelle, and cellular interactions. These interactions synergistically contribute to ensuring the effective operation of this crucial process. Additionally, this process entails the selective removal of compromised or dysfunctional mitochondria from the cell, intricately entwined with mitochondrial function and autophagy.

## 3. Evidence for mitochondrial dysfunction in depression

Mitochondria serve as semi-autonomous organelles in eukaryotic cells. They are pivotal for various cellular functions and signaling cascades (Spinelli and Haigis, 2018; Belenguer et al., 2019). These organelles are also the primary sites for aerobic respiration and generate ATP to support essential neuronal processes such as neurogenesis, neurotransmission, and synaptic plasticity (Kann and Kovacs, 2007; Rangaraju et al., 2014). In the brain, mitochondria are instrumental in regulating neural activity, plasticity, and behavioral adaptation (Grimm and Eckert, 2017; Todorova and Blokland, 2017; Angelova and Abramov, 2018). Mitochondrial damage not only fails to meet the energy demands of cells, but also impairs neuronal communication and cellular resilience, potentially leading to mood disorders and mental illness (Quiroz et al., 2008; Rezin et al., 2009). This is primarily manifested by alterations in mitochondrial structure, decreased MMP levels, excessive production of ROS, reduced ATP synthesis capacity, mtDNA damage and other factors.

Preclinical and clinical data provide evidence indicating that there exists dysfunction in the mitochondria of individuals with depression as well as in animals displaying behavior similar to depression. Reports have highlighted compromised ATP production and mtDNA issues in depressed patients (Czarny et al., 2018). Specifically, these patients have exhibited diminished respiratory indices, encompassing regular respiration, uncoupled respiration, spare respiratory capacity, coupling efficiency and ATP conversion rates (Karabatsiakis et al., 2014). Meanwhile, their mtDNA copy numbers have proven to be notably higher than those of healthy individuals (Ryan et al., 2023). Furthermore, depression has been associated with increased levels of mtROS and enhanced amounts of mtDNA (Cai et al., 2015; Tripathi et al., 2021), suggesting that mitochondrial dysfunction may lead to energy depletion in the brain and contribute to the development of depression (Morava et al., 2010; Gardner and Boles, 2011).

Likewise, mitochondrial harm was noted in both afflicted animals and cells. Several animal models have been developed to mimic the depressive symptoms of patients with depression, and chronic unpredictable mild stress (CUMS), chronic restraint stress (CRS) and chronic social defeat stress (CSDS) are usually used to simulate stress-induced depression. Rodents with depression-like behaviors display increased immobility time in tail suspension test and forced swimming test (despair behavior), and decreased sucrose preference (namely anhedonia). Mice with depression, triggered by either CUMS or CMS, showed a decrease in MMP levels and a suppression of the rate of mitochondrial respiration. Moreover, their mitochondria demonstrated structural anomalies like enlargement, vacuolar degeneration, irregular inner cristae formation, or even dissolution/disappearance (Gong et al., 2011; Yuan et al., 2019; Wang et al., 2022). In addition to this, the level of ROS was growing in CUMS induced mice and microglia induced by LPS and ATP. Furthermore, the MMP was reduced in microglia induced by LPS and ATP. Decreased ATP levels and increased mtDNA copy number were also seen in Dex-induced mice (Arioz et al., 2019; Li et al., 2020; Shen et al., 2021; Wang et al., 2023).

Taken together, mitochondrial dysfunction results in escalated oxidative stress, mtDNA damage or deletions, alterations in mitochondrial fusion/fission and morphology, ultimately leading to neuronal cell demise. The process of mitophagy stands as a pivotal mechanism for upholding mitochondrial quality control through the removal of aged, dysfunctional, damaged or excessive mitochondria (Palikaras et al., 2018). This mechanism also serves to delay the onset of mitochondrial dysfunction instigated by oxidative stress and lessen the accumulation of mtDNA and ROS. In doing so, it ensures the preservation of the typical structure and function within the mitochondrial network, facilitating cellular equilibrium. Deviations in mitophagy can culminate in the accumulation of impaired mitochondria, thereby fostering depression.

## 4. Evidence for autophagy abnormalities in depression

Autophagy is a vital cellular mechanism present in eukaryotic cells. It is responsible for transporting damaged organelles and malformed proteins to lysosomes for degradation, thereby maintaining cellular homeostasis (Ulrich et al., 2020). Altered autophagy-related signaling pathways have been identified in patients and animal models of depression. The mammalian target of rapamycin (mTOR) serves as a critical regulator of autophagy (Winden et al., 2018). Its phosphorylated form (p-mTOR) indicates activation of the autophagic pathway (Wander et al., 2011; Fiorini et al., 2013). Autopsy findings revealed a significant reduction in the expression of mTOR and its downstream effectors (p70S6K, eIF4B, and p-eIF4B) within the prefrontal cortex of depressed patients compared to age-matched healthy controls (Jernigan et al., 2011). Autophagy is accompanied by changes in related proteins, including Beclin-1, LC3, and P62 (Wei et al., 2008; Choi et al., 2013; Cai et al., 2015; Ranjan and Pathak, 2016). The expression of autophagy genes LC3B, ATG12 and Beclin-1 was upregulated in peripheral blood mononuclear cells of depressed patients (Alcocer-Gomez et al., 2017).

In the hippocampus of depression model rats, autophagy was activated, leading to reduced p-mTOR and P62 expression, and a notable increase in Beclin-1 expression (Ning et al., 2023). Depressed rats induced by CUMS displayed elevated levels of Beclin-1 and LC3BII/I in the CA1 hippocampal region, along with increased autophagosomes observed through electron microscopy, indicating autophagy activation (Hao et al., 2013; Zhang Z. et al., 2020). On the contrary, the autophagy process was inhibited in both Lipopolysaccharide (LPS)-induced mice and astrocytes. The size and number of autophagosomes were elevated, while the LC3BII/I ratio and Beclin-1 expression dramatically rose. In contrast, P62 expression notably decreased (Li et al., 2021).

Autophagy has been confirmed to play a role in the pathogenesis and progression of depression, and certain antidepressants exert their therapeutic effects by modulating autophagic flux. Oridonin, a diterpene compound isolated from *Rabdosia rubescens* with diverse biological properties (Liu and Du, 2020), exhibits potential in alleviating depression-like behaviors. It has been observed to increase the sucrose preference rate and decrease immobility time in both the forced swimming test (FST) and tail suspension test (TST) in mice. This effect might be attributed to the upregulation of autophagy levels, evident from an elevated LC3BII/I ratio and Beclin-1 protein expression, while P62 protein was downregulated in the brains of depressed mice. A similar effect was also observed in LPS-induced astrocytes. Importantly, the autophagy-inducing agent Rapamycin synergistically enhanced oridonin-mediated upregulation of LC3BII/I, Beclin-1, and P62 protein expression. Conversely, the autophagy inhibitor 3-Methyladenine abrogated oridonin-induced promotion of autophagy (Li et al., 2021).

The tricyclic antidepressant amitriptyline can impede autophagic flux by disrupting the fusion of autophagosomes and lysosomes, possibly due to LC3BII accumulation induced by amitriptyline (20 μM), with or without NH_4_Cl (an autophagosome-lysosome fusion inhibitor). A significant portion of LC3B and P62 immunoreactivities were co-localized, but not with LAMP2 (Kwon et al., 2020). Concurrently with LC3BII induction, there was a subtle increase in Beclin-1 expression observed following treatment with amitriptyline or the selective serotonin re-uptake inhibitor citalopram (Zschocke et al., 2011). Ketamine exhibited rapid-onset effects in the treatment of depression, inducing autophagy in microglia by upregulating LC3B levels and downregulating P62 protein expression. Additionally, the ketamine-induced increase in autophagy can be impeded by bafilomycin A1, an autophagy inhibitor (Lyu et al., 2022).

Both individuals and animal models with depression have been observed to display altered autophagy-associated signaling. Autophagy is a crucial cellular mechanism for eliminating damaged or dysfunctional components from cells, and its disruption can result in the accumulation of toxic substances and other harmful materials within cells. This accumulation could potentially contribute to the development of various diseases, including depression. Mitophagy, a form of selective autophagy, can also be influenced by changes in autophagy levels.

## 5. Evidence for impaired mitophagy in depression

### 5.1. Impaired mitophagy in MDD patients

Clinical research indicates that changes in mitophagy-related protein levels may relate to depression severity. Patients with depression might experience impaired mitochondria clearance, seen through higher PINK1, P62, and LC3B levels in peripheral blood nuclear cells, and lower Parkin levels (Scaini et al., 2022). The mRNA levels of PINK1, NIX, and LC3A were significantly lower in the blood of MDD patients (Weixing, 2019; Lu et al., 2023). The 18 kDa translocator protein (TSPO) has gained increased attention for its role as a crucial rate-limiting step in neurosteroidogenesis and its potential implications in the pathophysiology of stress response and related disorders (Beurdeley-Thomas et al., 2000; Pinna and Rasmusson, 2012). TSPO hinders mitophagy downstream of the PINK1/Parkin pathway by impeding crucial protein ubiquitination, and its function depends on the voltage-dependent anion channel (VDAC1; Gatliff et al., 2014). Clinical studies have demonstrated significantly elevated the TSPO density by distribution volume in the serum of patients experiencing extreme depressive episodes (Setiawan et al., 2015).

In summary, the impaired mitophagy observed in patients with depression is associated with anomalies in transcriptional processes and corresponding protein expression. Although a few clinical studies have explored this relationship, existing data are not sufficient. Additional indicators related to patient conditions are needed for further validation. Importantly, elucidating the role of mitochondrial autophagy in depression may open avenues for new therapeutic strategies for patients suffering from this condition. By conducting more extensive studies on the connection between mitochondrial autophagy and depression, researchers could acquire new insights into optimal treatment and management approaches for affected patients.

### 5.2. Impaired mitophagy in MDD models

Disruption of mitophagy, the selective removal of damaged mitochondria, may significantly contribute to depression-like behavior in animals, as indicated by several studies. Studies have revealed inhibited mitophagy levels in animal models of depression induced by learned helplessness (LH) and social defeat stress (SDS). These models showed substantial decreases in the expression of key proteins involved in mitophagy such as TSPO, Parkin, VDAC1, and the autophagy initiator protein Beclin-1 (Li et al., 2016; Wei et al., 2020). Similarly, mitophagy suppression was observed in the hippocampus of rats induced with chronic CUMS. This was characterized by reduced protein and mRNA expression of mitophagy-related proteins PINK1 and Parkin, along with autophagy protein Beclin-1, while protein and mRNA levels of P62 were increased (Meng, 2020). Jin et al. discovered that NIX-mediated mitophagy degradation was impaired in hippocampal neurons of CUMS-induced mice, leading to the accumulation of damaged mitochondria. This resulted in increased protein expressions of LC3BII/I, P62, and TOM20. Notably, NIX protein expression was prominently lower in the CUMS group compared to controls, whereas no differences were observed for Parkin protein (Jin et al., 2023). Similarly, the mRNA expression of NIX and LC3A was downregulated in the blood of mice induced by LPS and CSDS, while OPTN and NDP52 proteins remained unaffected in CSDS (Lu et al., 2023).

MDD is an emotional disorder associated with stress, and prolonged exposure to stress heightens susceptibility to depression (CONVERGE consortium, 2015). The social defeat stress model is commonly employed in depression research (Suzuki et al., 2021). Mitophagy and autophagy activation were observed in the hippocampus following social defeat stress, leading to increased expression of Beclin-1, ATG5, LC3B II, P62, LAMP2, PINK1, and Parkin, with the exception of TOM20, which showed reduced levels (Guo et al., 2022). Diabetes-related depression (DD) is a major complication of diabetes, and DD rats exhibited behaviors similar to depression, such as increased immobility time in the FST. Mitophagy disorders occurred in the DD rats, which results in an upregulation of related proteins LC3B, Beclin-1, and Parkin, while a downregulation of P62 and mTOR expression (Liu et al., 2021).

In BV2 cells stimulated by LPS and ATP, impaired mitophagy degradation led to elevated levels of LC3BII and prominently reduced levels of P62 in both the cytoplasm and mitochondria. Concurrently, mitochondrial levels of PINK1 and Parkin were notably decreased, while the colocalization of P62 and TOM20 through immunofluorescence increased (Han et al., 2021). Similarly, mitophagy levels were diminished in corticosterone (CORT)-induced HT22 cells, resulting in the accumulation of damaged mitochondria. This was accompanied by increased protein expressions of LC3BII/I, P62, and TOM20 (Jin et al., 2023).

Overall, these findings suggest a significant disruption in the process of selective autophagy targeting damaged mitochondria in various animal strains and cellular models. Abnormal expression of key proteins such as PINK1, Parkin, LC3B, and P62 indicates a breakdown in the cellular machinery responsible for clearing damaged mitochondria. This disruption may have profound implications for cellular health and function, providing crucial insights into the impact of impaired mitochondrial autophagy on overall cellular well-being. Moreover, these findings may have broader implications for conditions linked to impaired mitophagy, such as depression (Table 1).

**Table 1:** The alterations in mitophagy observed in depression models.

Species	The model of animals or cells	Sample Source	Experimental approaches/methods	Molecular modifications	Expected phenotypic manifestations	Refs.
Human		Peripheral blood mononuclear cells	WB	↑PINK1, P62, and LC3B proteins ↓Parkin protein	↓The mitophagy degradation process	Scaini et al. (2022)
			RT-qPCR	↓PINK1 mRNA	↓Mitophagy level	Weixing (2019)
		Peripheral blood	RT-qPCR	↓NIX and LC3A mRNA	↓NIX-mediated mitophagy	Lu et al. (2023)
		Serum	[^18^F] FEPPA PET	↑TSPO VT	ND	Setiawan et al. (2015)
Animal	LH mice	The mesencephalon of mice	WB	↓TSPO, PINK1, VDAC1, and Beclin-1 proteins ↑Parkin protein	↓Mitophagy level	Li et al. (2016)
				↓TSPO, Parkin, VDAC1, and Beclin-1 proteins	↓TSPO-mediated mitochondrial dysregulation	Wei et al. (2020)
	SDS mice					
	CUMS rat	Hippocampus	WB and RT-qPCR	↓PINK1, Parkin, Beclin-1 mRNA and proteins ↑ P62 mRNA and protein	↓PINK1/Parkin-mediated mitophagy	Meng (2020)
	CUMS mice		WB	↑LC3BII/ I ratio, P62, TOM20 proteins ↓NIX protein - Parkin protein	↓NIX-mediated mitophagy degradation	Jin et al. (2023)
	LPS mice	Blood and mPFC	WB and RT-qPCR	↓NIX and LC3A mRNA - OPTN protein -NDP52 protein	↓NIX-mediated mitophagy	Lu et al. (2023)
	CSDS mice					
	SDS mice	Hippocampus	WB	↑Beclin-1, ATG5, LC3BII, P62, LAMP2, PINK1, and Parkin proteins ↓TOM20 protein	↑PINK1/Parkin-mediated mitophagy	Guo et al. (2022)
	DD rat			↑LC3B, Beclin-1, and Parkin proteins ↓P62, mTOR protein	↑Mitophagy activation	Liu et al. (2021)
Cell	LPS and ATP-induced BV2 cell		WB and IF	Cytoplasm: ↓LC3BII, ↑P62 proteins Mitochondria: ↓LC3BII, PINK1, Parkin, and ↑P62 proteins, ↑Immunofluorescence colocalization of P62 with TOM20	↓The mitophagy degradation process	Han et al. (2021)
	CORT-induced HT22 cell		WB	↑LC3BII/ I ratio, P62, TOM20 proteins, ↓NIX protein - Parkin protein	↓NIX-mediated mitophagy degradation	Jin et al. (2023)

CSDS, chronic social defeat stress; CUMS, chronic unpredictable mild stress; CORT, corticosterone; DD, diabetes-related depression; IF, immunofluorescence; LH, learned helplessness; RT-qPCR, real-time polymerase chain reaction; SDS, social defeat stress; TSPO VT, translocator protein density by distribution volume; WB, western blot. ↑, increased; ↓, decreased; or, unchanged; ND, not determined.

### 5.3. Investigating the impaired mitophagy of depression for drug research

#### 5.3.1. The effect of Chinese herbal medicine on mitophagy

Chinese herbal medicine has gained recognition for its efficacy in alleviating symptoms of depression (Butler and Pilkington, 2013). Its antidepressant effects are believed to be associated with the regulation of mitophagy levels. Wuling powder is a Chinese herbal medicine extracted from Xylaria Nigripes (Kl.) Sacc using modern fermentation technology, and was approved by China State Food and Drug Administration (Authorized Document Number: Z19990048 in Chinese medicine) for treating insomnia in 1999. It has been shown to exhibit antidepressant effects in multiple behavioral tests, with increased success rates in shuttle box escape and shortened latencies in novelty suppressed feeding test (NSF) and FST immobility time when administered at a dose of 500 mg/kg to LH mice. Wuling powder also enhanced damaged mitochondria elimination and alleviated mitophagy impairment by elevating the expression of mitophagy-related proteins TSPO, VDAC1, PINK1, and Beclin-1 in the brain, while reducing Parkin (Li et al., 2016). Xiao Jianzhong Decoction that can be used in the treatment of neurasthenia and insomnia in clinic is derived from the “treatise on febrile and miscellaneous diseases” of Zhang Zhongjing in the Eastern Han Dynasty, and has a long history of application. Xiao Jianzhong Decoction contains active compounds including paeoniflorin, cinnamic aldehyde and liquiritin that exhibit significant antidepressant effects. Administration of Xiao Jianzhong decoction effectively alleviated depression-like behaviors in CUMS-induced rats as evidenced by reduced immobility time in FST and increased total distance and time spent in open field test (OFT). This may be particularly pertinent for the upregulation of mitophagy mediated by PINK1/Parkin in the hippocampus of CUMS-induced rats through Xiao Jianzhong decoction, as evidenced by significant increases in protein expression and mRNA levels of PINK1, Parkin, and Beclin-1, along with notable reductions in P62 protein and mRNA levels (Meng, 2020). *Piper laetispicum* C. DC, a Chinese herbal remedy, demonstrated potential for alleviating depressive disorders. Clinical trials indicated that the aqueous extract of *Piper methysticum* can improve depression symptoms. G11-5 [3-(3,4-methylenedioxy-5-trifluoromethyl phenyl)-2E-propenoic acid isobutyl amide], a compound derived from the active ingredients of *Piper laetispicum* C. DC plants, has higher lipid solubility, but its toxicity still needs to be further studied. G11-5 can improve depression-like behavior in LH and SDS mice, and leads to an increased success rate for electric shock escape and greater total distance traveled during OFT movement, as well as reduced FST immobility time. Furthermore, G11-5 regulated mitophagy levels and increasing the expression of TSPO, Parkin, VDAC1, and autophagy promoter Beclin-1 in the brain of LH mice (Wei et al., 2020).

Microglia are the resident immune surveillance cells of the central nervous system (von Bernhardi et al., 2016). The results of previous experiments have shown that inflammation mediated by activated microglia plays a crucial role in the development of MDD (Song and Colonna, 2018). Quercetin, a natural flavonoid with anti-inflammatory and antioxidant properties. It can prevent neuronal damage by promoting mitophagy and inhibiting mtROS-mediated activation of the NLRP3 inflammasome in microglia. Treatment with quercetin effectively restores impaired mitophagy in LPS-and ATP-stimulated BV2 cells, as evidenced by the upregulated expression levels of LC3BII, PINK1, and Parkin, along with the downregulated levels of P62 protein, and reduced co-localization of P62 with TOM20 observed through immunofluorescence (Han et al., 2021).

Baicalin, the primary bioactive constituent of *Scutellaria baicalensis,* has demonstrated antidepressant-like effects in various rodent models (Li et al., 2015). In CUMS-induced mice, intragastric administration of baicalin (20 mg/kg) for 4 weeks effectively ameliorated depression-like behaviors by markedly increasing the sucrose preference rate and reducing the immobility time in TST. Through investigating its molecular mechanism, baicalin was found to promote the elimination of damaged mitochondria in mice hippocampal neurons and enhance mitophagy levels mediated by NIX. This process ameliorates aberrant expression of LC3B II/I, P62, NIX, and TOM20 proteins. Additionally, baicalin markedly improved the expression of LC3BII/I, P62, and TOM20 while reducing NIX protein levels in CORT-induced HT22 cells (Jin et al., 2023).

During the course of antihypertensive treatment, *Morinda officinalis* oligosaccharides, a natural extract derived from the root of *Morinda officinalis*, have demonstrated antidepressant properties (Xu et al., 2017; Zhang et al., 2018). The depression-like behavior of CUMS-induced rats can be alleviated through the administration of *Morinda.* This intervention increases the sucrose preference rate and reduces the immobility time of rats in FST and TST. *Morinda officinalis* oligosaccharides were found to enhance autophagic flux and mitophagy in LPS-induced astrocytes, leading to a reduction in P62 levels and an increase in LC3B expression. This process facilitated the translocation of Parkin to the mitochondria and resulted in TOM20 degradation, ultimately reversing ectopic expression of LC3B and P62 (Yang et al., 2023).

In recent times, there has been a growing interest in the potential therapeutic effects of herbal remedies for depression. Research suggests that specific Chinese herbal medicines can effectively modulate levels of mitophagy, thereby positively influencing mood and alleviating depressive symptoms.

#### 5.3.2. The effect of classic antidepressants on mitophagy

Antidepressant pharmacotherapy is an efficacious intervention for depression (Cho et al., 2016), with monoamine oxidase inhibitors, tricyclic antidepressants, selective serotonin reuptake inhibitors (SSRIs), and serotonin and norepinephrine reuptake inhibitors being commonly prescribed agents (Xu et al., 2010). Fluoxetine, a pioneer of the SSRI class, has gained widespread used for its significant clinical efficacy and favorable safety profile (Perez-Caballero et al., 2014; Micheli et al., 2018; Shu et al., 2019; Hetrick et al., 2021). In a study involving CUMS-induced mice, fluoxetine was found to enhance NIX-mediated mitophagy by reducing the LC3BII/I and P62 expression while increasing NIX expression, without affecting Parkin levels (Lu et al., 2023). Additionally, the level of mitophagy was promoted by regulating the levels of mitophagy-related proteins such as TSPO, VDAC1, PINK1, and Beclin-1 in LH mice brains. However, Parkin expression was downregulated (Li et al., 2016). Astrocytes, abundant cells in the central nervous system, play a pivotal role in the pathogenesis of MDD due to their prevalence and substantial volume in the cortex and hippocampus (Kong et al., 2014; Pekny et al., 2016). Given their role in metabolic support and brain function regulation, efficient mitophagy is crucial to meet their high energy demands (Hertz et al., 2007). Fluoxetine enhances the removal of damaged mitochondria and promotes autophagic flux in astrocytes from CMS mice and primary cultured mouse astrocytes. This is evidenced by an increase in the LC3BII/I ratio and a decrease in P62 protein expression. Furthermore, fluoxetine induces mitophagy in primary astrocytes by downregulating cytoplasmic Parkin and mitochondrial TOM20 expression levels while upregulating mitochondrial Parkin expression (Shu et al., 2019). Citalopram, an SSRI, exerts protective effects on mitophagy in a transgenic mouse model of Alzheimer’s disease (AD) expressing amyloid precursor protein (APP). Treatment with citalopram sensibly upregulates the mRNA levels of LC3B, ATG5, PINK1, Beclin-1 and BNIP3L in APP mice. However, it leads to significant downregulation of the expression of proteins such as PINK1, ATG5, ATG7, P62 and LC3BII/I (Reddy et al., 2021a). Moreover, it augmented the autophagic and mitophagy activity of mAPP-HT22 cells, significantly elevating mRNA levels of LC3B, ATG5, Beclin-1, PINK1 and BNIP3L while reducing protein expressions of PINK1, LC3BII, ATG5, ATG7 and P62 (Reddy et al., 2021b).

Ketamine, a frequently utilized intravenous anesthetic and analgesic in clinical practice, has recently been indicated to possess distinct advantages in antidepressant research owing to its rapid-onset antidepressant effect (Chen-Li et al., 2022). In mice exhibiting depression-like behavior induced by LPS, ketamine effectively enhanced their sucrose preference rate, reduced immobility time in FST and TST tests, and decreased feeding latency in NSFT. The LPS-induced blockage of BV2 cells’ autophagic flux was reversed and early mitophagy activation was upregulated with the treatment of ketamine, which elevated the mRNA levels and protein expressions of PINK1, Beclin-1, and ATG5. Additionally, LC3BII/I and LAMP1 levels in LPS-injured BV2 cells were observed to increase, while the expression of P62 protein decreased following treatment with ketamine (Wu et al., 2022). Lu et al. proposed that NIX-mediated mitophagy could potentially serve as an antidepressant mechanism for ketamine. The study revealed that ketamine rescued TNFα-induced behavioral despair, as evidenced by a reduction in immobility time in the TST and FST, without impacting locomotion activity. Moreover, ketamine mitigated TNF-α-induced NIX deficiency in the mPFC and reversed the reduction of Beclin-1 and LC3BII proteins in the mPFC of TNF-α-treated mice. However, the knockout of NIX prevented the increase in stress-coping behaviors induced by ketamine in TNF-α-treated mice, while locomotion activity remained unaffected (Lu et al., 2023).

In simple terms, both classic and rapid antidepressants have demonstrated promising outcomes in treating depression and related illnesses like AD due to their ability to regulate mitophagy levels (Table 2).

**Table 2:** Modulation of mitophagy levels in the depression model by pharmacological interventions.

Drug type		Models	Administration	Route	Sample Source	Experimental approaches/methods	Behavioral changes	Molecular mechanisms	Expected phenotypic manifestations	Refs.
Wuling powder	Chinese medicine compound	LH mice	500 mg/kg for 2 weeks	Gavage administration	The mesencephalon of mice	WB	Shuttle box: ↓Number of escape failures, ↓Average escape latency NSFT: ↓Feeding latency FST: ↓Immobility time	↑TSPO, PINK1, VDAC1, and Beclin-1 proteins ↓Parkin protein	↑Mitophagy level	Li et al. (2016)
Xiao Jianzhong Decoction		CUMS rat	3,600 mg/kg,7,200 mg/kg, and 14,400 mg/kg for 3 weeks		Hippocampus	WB and RT-qPCR	FST: ↓Immobility time OFT: ↑Total distance and total time of exercise	↑mRNA and protein levels of PINK1, Parkin, and Beclin-1 ↓ P62 mRNA and protein	↑PINK1/Parkin-mediated mitophagy	Meng (2020)
G11-5	Plant derivatives	LH and SDS mice	5 mg/kg, 10 mg/kg, and 20 mg/kg for 2 weeks	ND	The mesencephalon of mice	WB	Shuttle box: ↑Escape success rate FST: ↓Immobility time OFT: ↑Total distance of exercise	↑TSPO, Parkin, VDAC1, and Beclin-1 proteins	↓TSPO-mediated mitochondrial dysregulation	Wei et al. (2020)
Quercetin	Chinese herbal medicine monomer	LPS and ATP-stimulated BV2 cell	30/100 μM for 1 h			WB and IF		↑LC3BII, PINK1, Parkin protein ↓P62 protein ↓Immunofluorescence colocalization of P62 with TOM20	↑The mitophagy degradation process	Han et al. (2021)
Baicalin		CUMS mice	20 mg/kg for 4 weeks	Gavage administration	Hippocampus		SFT: ↑Sucrose preference rate TST: ↓Immobility time	↓LC3BII/I ratio, P62, TOM20 protein ↑NIX protein	↑NIX-mediated mitophagy degradation	Jin et al. (2023)
		CORT-induced HT22 cell	4 μM for 1 h			WB				
Morinda officinalis oligosaccharides		CUMS rat	100 mg/kg for 4 weeks	Gavage administration	Brain	WB and TEM	SFT: ↑Sucrose preference rate FST: ↓Immobility time TST: ↓Immobility time	↓Total protein P62, Cytoplasmic Parkin and Mitochondrial TOM20 protein ↑LC3BII/I ratio ↓Mitochondrial damage such as swollen mitochondria, adventitia rupture, cavitation	↑Autophagic flux and mitophagy level	Yang et al. (2023)
		LPS-induced astrocytes cell	2.5 and 5 mg/mL for 24 h							
Fluoxetine	SSRIs	CUMS mice	20 mg/kg for 4 weeks	Gavage administration	Hippocampus	WB	SFT: ↑Sucrose preference rate TST: ↓Immobility time	↓LC3BII/I ratio, P62, TOM20 protein ↑NIX protein	↑NIX-mediated mitophagy degradation	Lu et al. (2023)
		LH mice	10 mg/kg for 2 weeks		The mesencephalon of mice		Shuttle box: ↑Escape success rate FST: ↓Immobility time OFT: ↑Total distance of exercise	↑TSPO, Parkin, VDAC1, Beclin-1 proteins	↓TSPO-mediated mitochondrial dysregulation	Li et al. (2016)
		CMS mice	10 mg/kg for 4 weeks		Hippocampus	WB and TEM	FST: ↓Immobility time TST: ↓Immobility time	↑LC3BII/I ratio ↓ P62 protein ↓Mitochondrial damage	↑The clearance of damaged mitochondria and unblocked autophagic flux	Shu et al. (2019)
		Primary cultured mice astrocytes cell	10 μM for 1 h					Total:↑LC3BII/I ratio↓ P62 protein Cytoplasm: ↓Parkin protein Mitochondria: ↓TOM20 protein, ↑Parkin protein	↑Mitophagy induced	
Citalopram		APP mice	20 mg/kg for 4 weeks	Intraperitoneal injection	Cerebral cortex	WB and RT-qPCR		↑LC3B, ATG5, PINK1, Beclin-1, and BNIP3L mRNA ↑PINK1, ATG5, ATG7, P62, LC3BI, and LC3BII proteins	↑Mitophagy activation	Reddy et al. (2021a)
		mAPP-HT22 cell	20 μM for 24 h							Reddy et al. (2021b)
Ketamine	N-methyl-d-aspartate receptor antagonist	LPS mice	10 mg/kg for 24 h				SFT: ↑Sucrose preference rate FST: ↓Immobility time TST: ↓Immobility time NSFT: ↓Feeding latency		↑Early mitophagy activation and autophagy flux	Wu et al. (2022)
		LPS-induced BV2 cell				WB, RT-qPCR and mRFP-GFP-LC3		↑PINK1, Beclin-1, ATG5 mRNA and proteins, LC3BII/I ratio, LAMP1 protein ↓ P62 protein ↑mRFP-GFP-labeled LC3		
		TNF-α mice	10 mg/kg was administered after TNFα treatment for 30 min	Intraperitoneal injection	mPFC	WB	FST: ↓Immobility time TST: ↓Immobility time	↑NIX, Beclin-1, and LC3BII proteins	↑NIX-mediated mitophagy	Lu et al. (2023)

CMS, chronic mild stress; CUMS, chronic unpredictable mild stress; CORT, corticosterone; FST, forced swimming test; IF, immunofluorescence; LH, learned helplessness; LPS, lipopolysaccharide; OFT, open field test; RT-qPCR, real-time polymerase chain reaction; SDS, social defeat stress; SSRIs, selective serotonin reuptake inhibitors; SFT, sucrose preference test; TNF-α, tumor necrosis factor-α; TST, tail suspension test; TEM, transmission electron microscopy; WB, western blot; mRFP-GFPLC3 probes, the tandem fluorescent-tagged LC3 probe monitoring autophagic flux based on different pH stability of mRFP; h, hour. ↑, increased; ↓, decreased; or, unchanged; ND, not determined.

## 6. Conclusion and prospects

Depression, a chronic illness that affects millions of people worldwide, has undergone extensive research in recent years. Although some progress has been made, current treatment options remain limited and often fail to adequately alleviate symptoms for many patients with depression. Therefore, an imperative demand for innovative therapeutic approaches exists. Based on the current research progress, we believe that restoring the level of mitophagy may be an innovative approach to improve the therapeutic effect of depression. Multiple lines of evidence reflect that mitochondrial dysfunction is linked to depression in various regions of the brain (Bansal and Kuhad, 2016; Marx et al., 2021; Hollis et al., 2022; Khan et al., 2023). Patients with mitochondrial diseases, mutations, and polymorphisms in mtDNA may undergo mood changes, cognitive function alterations, psychosis, and anxiety (Anglin et al., 2012a,b; Mancuso et al., 2013). Mitophagy is a cellular process that eliminates damaged mitochondria, effectively regulating mitochondrial quality and quantity to uphold cellular homeostasis. The regulation of mitophagy holds promising applications in the investigation and clinical management of neurological disorders like Parkinson’s disease (PD) and AD (Kerr et al., 2017; Lizama and Chu, 2021). The regulation and functions of mitophagy share many similarities across PD, AD, and MDD. Furthermore, the observed alterations in mitophagy and mitochondrial function in depression propose that targeting mitophagy could be a promising therapeutic avenue.

Recent studies have shown that mitophagy plays a role in the development of depression. The mitochondrial damage caused by impaired mitophagy affects the process of mitochondrial ATP production, which impairs neuroplasticity and then negatively affects the development of depression (Bertholet et al., 2016). Mitophagy can also inhibit microglia-mediated neuroinflammation by suppressing the activation of inflammasomes, thereby attenuating depressive symptoms (Sprague and Khalil, 2009; Su et al., 2017; Taene et al., 2020). This review succinctly encapsulates recent advancements in linking mitophagy failure to the pathogenesis of MDD. Aberrant expression of the mitophagy marker PINK1 and related proteins in individuals with clinical depression underscores that mitophagy failure could potentially serve as a causal factor for MDD. Preclinical depression models also substantiate this hypothesis. These harmonious findings improve the concept that salvaging mitophagy in MDD might constitute a promising therapeutic strategy. Our review highlights that several antidepressants and effective compounds derived from Chinese herbal medicine, such as fluoxetine, ketamine, and baicalin, which have demonstrated significant amelioration of abnormal pathological and behavioral manifestations in MDD models through the induction of mitophagy. Despite some progress in exploring the relationship between mitochondrial autophagy and depression, an urgent necessity persists for a more comprehensive investigation into the evolution of this process during the progression of MDD. To gain a comprehensive understanding, it is necessary to collect more clinical data and conduct extensive preclinical studies. Only then can we hope to unravel the complex interplay between mitochondrial autophagy and depression, paving the way for potentially life-changing novel therapeutic interventions.

## Author contributions

WX: writing, review and editing – original draft. WG, YG, FX, LD, SF, LF, YZ, and YH: investigation. YZ: supervision. XX and XP: project administration. All authors contributed to the article and approved the submitted version.

## Figures and Tables

**Table:** 

AD	Alzheimer’s disease
APP	Amyloid precursor protein
ATP	Adenosine triphosphate
Bcl-2	B-cell leukemia/lymphoma 2
BNIP3	CL-2 and adenovirus E1B 19-kDa interacting protein 3
Ca2+	Calcium ions
CMS	Chronic mild stress
CORT	Corticosterone
CSDS	Chronic social defeat stress
CUMS	Chronic unpredictable mild stress
DD	Diabetes-related depression
FST	Forced swimming test
FUNDC1	FUN14 domain-containing 1
GABARAP	Gamma-aminobutyric acid receptor-associated protein
h	Hour
IF	Immunofluorescence
IMM	Inner mitochondrial membrane
LH	Learned helplessness
LIR	LC3-interacting region
LPS	Lipopolysaccharide
MDD	Major depressive disorder
MMP	Mitochondrial membrane potential
mRFP-GFPLC3 probes	The tandem fluorescent-tagged LC3 probe monitoring autophagic flux based on different pH stability of mRFP
mtROS	Mitochondrial ROS
NDP52	Nuclear dot protein 52 kDa
NIX	B-cell leukemia/lymphoma 2 and adenovirus E1B 19-kDa interacting protein 3-like
NSF	Novelty suppressed feeding test
OFT	Open field test
OMM	Outer mitochondrial membrane
OPTN	Optineurin
PD	Parkinson’s disease
PINK1	PTEN-induced putative kinase 1
p-mTOR	Phosphorylated mTOR
p-S65-Ub	Phosphorylate ubiquitin at S65
ROS	Reactive oxygen species
RT-qPCR	Real-time polymerase chain reaction
SDS	Social defeat stress
SFT	Sucrose preference test
SQSTM1	Sequestosome 1 also known as P62
SSRIs	Selective serotonin reuptake inhibitors
TEM	Transmission electron microscopy
TNF-α	Tumor necrosis factor-α
TSPO	Translocator protein
TST	Tail suspension test
VDAC1	Voltage-dependent anion channel
WB	Western blot

## References

[ref1] Alcocer-Gomez E. Casas-Barquero N. Nunez-Vasco J. Navarro-Pando J. M. Bullon P. (2017). Psychological status in depressive patients correlates with metabolic gene expression. CNS Neurosci. Ther. 23, 843–845. doi: 10.1111/cns.12755, PMID: 28879683 PMC6492754

[ref2] Allen J. Romay-Tallon R. Brymer K. J. Caruncho H. J. Kalynchuk L. E. (2018). Mitochondria and mood: mitochondrial dysfunction as a key player in the manifestation of depression. Front. Neurosci. 12:386. doi: 10.3389/fnins.2018.00386 29928190 PMC5997778

[ref3] Angelova P. R. Abramov A. Y. (2018). Role of mitochondrial ROS in the brain: from physiology to neurodegeneration. FEBS Lett. 592, 692–702. doi: 10.1002/1873-3468.12964, PMID: 29292494

[ref4] Anglin R. E. Garside S. L. Tarnopolsky M. A. Mazurek M. F. Rosebush P. I. (2012a). The psychiatric manifestations of mitochondrial disorders: a case and review of the literature. J. Clin. Psychiatry 73, 506–512. doi: 10.4088/JCP.11r07237 22579150

[ref5] Anglin R. E. Tarnopolsky M. A. Mazurek M. F. Rosebush P. I. (2012b). The psychiatric presentation of mitochondrial disorders in adults. J. Neuropsychiatry Clin. Neurosci. 24, 394–409. doi: 10.1176/appi.neuropsych.11110345 23224446

[ref6] Arioz B. I. Tastan B. Tarakcioglu E. Tufekci K. U. Olcum M. Ersoy N.. (2019). Melatonin attenuates LPS-induced acute depressive-like behaviors and microglial NLRP3 Inflammasome activation through the SIRT1/Nrf 2 pathway. Front. Immunol. 10:1511. doi: 10.3389/fimmu.2019.01511 31327964 PMC6615259

[ref7] Ashrafi G. Schwarz T. L. (2013). The pathways of mitophagy for quality control and clearance of mitochondria. Cell Death Differ. 20, 31–42. doi: 10.1038/cdd.2012.81, PMID: 22743996 PMC3524633

[ref8] Bansal Y. Kuhad A. (2016). Mitochondrial dysfunction in depression. Curr. Neuropharmacol. 14, 610–618. doi: 10.2174/1570159X14666160229114755, PMID: 26923778 PMC4981740

[ref9] Belenguer P. Duarte J. M. N. Schuck P. F. Ferreira G. C. (2019). Mitochondria and the brain: bioenergetics and beyond. Neurotox. Res. 36, 219–238. doi: 10.1007/s12640-019-00061-7, PMID: 31152314

[ref10] Bertholet A. M. Delerue T. Millet A. M. Moulis M. F. David C. Daloyau M.. (2016). Mitochondrial fusion/fission dynamics in neurodegeneration and neuronal plasticity. Neurobiol. Dis. 90, 3–19. doi: 10.1016/j.nbd.2015.10.011, PMID: 26494254

[ref11] Beurdeley-Thomas A. Miccoli L. Oudard S. Dutrillaux B. Poupon M. F. (2000). The peripheral benzodiazepine receptors: a review. J. Neuro-Oncol. 46, 45–56. doi: 10.1023/a:1006456715525 10896204

[ref12] Bromet E. Andrade L. H. Hwang I. Sampson N. A. Alonso J. de Girolamo G.. (2011). Cross-national epidemiology of DSM-IV major depressive episode. BMC Med. 9:90. doi: 10.1186/1741-7015-9-90 21791035 PMC3163615

[ref13] Butler L. Pilkington K. (2013). Chinese herbal medicine and depression: the research evidence. Evid. Based Complement. Alternat. Med. 2013:739716. doi: 10.1155/2013/739716, PMID: 23476701 PMC3582075

[ref14] Cai N. Chang S. Li Y. Li Q. Hu J. Liang J.. (2015). Molecular signatures of major depression. Curr. Biol. 25, 1146–1156. doi: 10.1016/j.cub.2015.03.008, PMID: 25913401 PMC4425463

[ref15] Chen G. Cizeau J. Vande Velde C. Park J. H. Bozek G. Bolton J.. (1999). Nix and nip 3 form a subfamily of pro-apoptotic mitochondrial proteins. J. Biol. Chem. 274, 7–10. doi: 10.1074/jbc.274.1.7, PMID: 9867803

[ref16] Chen G. Han Z. Feng D. Chen Y. Chen L. Wu H.. (2014). A regulatory signaling loop comprising the PGAM5 phosphatase and CK2 controls receptor-mediated mitophagy. Mol. Cell 54, 362–377. doi: 10.1016/j.molcel.2014.02.034, PMID: 24746696

[ref17] Chen-Li D. Lui L. M. W. Rosenblat J. D. Lipsitz O. Teopiz K. M. Ho R.. (2022). Ketamine as potential treatment for postpartum depression: a narrative review. Ann. Clin. Psychiatry 34, 264–274. doi: 10.12788/acp.0082, PMID: 36282614

[ref18] Chipuk J. E. Bouchier-Hayes L. Green D. R. (2006). Mitochondrial outer membrane permeabilization during apoptosis: the innocent bystander scenario. Cell Death Differ. 13, 1396–1402. doi: 10.1038/sj.cdd.4401963, PMID: 16710362

[ref19] Cho H. Son S. J. Kim S. Park J. (2016). A randomized comparison of medication and cognitive behavioral therapy for treating depression in low-income Young minority women. Med. Sci. Monit. 22, 4947–4953. doi: 10.12659/MSM.902206, PMID: 27981956 PMC5189608

[ref20] Choi J. Jung W. Koo J. S. (2013). Expression of autophagy-related markers beclin-1, light chain 3A, light chain 3B and p 62 according to the molecular subtype of breast cancer. Histopathology 62, 275–286. doi: 10.1111/his.12002, PMID: 23134379

[ref21] Cipriani A. Furukawa T. A. Salanti G. Chaimani A. Atkinson L. Z. Ogawa Y.. (2018). Comparative efficacy and acceptability of 21 antidepressant drugs for the acute treatment of adults with major depressive disorder: a systematic review and network meta-analysis. Lancet 391, 1357–1366. doi: 10.1016/S0140-6736(17)32802-7, PMID: 29477251 PMC5889788

[ref22] Clark I. E. Dodson M. W. Jiang C. Cao J. H. Huh J. R. Seol J. H.. (2006). Drosophila pink 1 is required for mitochondrial function and interacts genetically with parkin. Nature 441, 1162–1166. doi: 10.1038/nature04779, PMID: 16672981

[ref23] Collaborators C.-M. D. (2021). Global prevalence and burden of depressive and anxiety disorders in 204 countries and territories in 2020 due to the COVID-19 pandemic. Lancet 398, 1700–1712. doi: 10.1016/S0140-6736(21)02143-7, PMID: 34634250 PMC8500697

[ref24] CONVERGE consortium (2015). Sparse whole-genome sequencing identifies two loci for major depressive disorder. Nature 523, 588–591. doi: 10.1038/nature14659, PMID: 26176920 PMC4522619

[ref25] Czarny P. Wigner P. Galecki P. Sliwinski T. (2018). The interplay between inflammation, oxidative stress, DNA damage, DNA repair and mitochondrial dysfunction in depression. Prog. Neuro-Psychopharmacol. Biol. Psychiatry 80, 309–321. doi: 10.1016/j.pnpbp.2017.06.036, PMID: 28669580

[ref26] Disease G. B. D. Injury I. Prevalence C. (2018). Global, regional, and national incidence, prevalence, and years lived with disability for 354 diseases and injuries for 195 countries and territories, 1990-2017: a systematic analysis for the global burden of Disease study 2017. Lancet 392, 1789–1858. doi: 10.1016/S0140-6736(18)32279-7, PMID: 30496104 PMC6227754

[ref27] Doblado L. Lueck C. Rey C. Samhan-Arias A. K. Prieto I. Stacchiotti A.. (2021). Mitophagy in human diseases. Int. J. Mol. Sci. 22:83903. doi: 10.3390/ijms22083903, PMID: 33918863 PMC8069949

[ref28] Fiorini C. Menegazzi M. Padroni C. Dando I. Dalla Pozza E. Gregorelli A.. (2013). Autophagy induced by p 53-reactivating molecules protects pancreatic cancer cells from apoptosis. Apoptosis 18, 337–346. doi: 10.1007/s10495-012-0790-6, PMID: 23238993

[ref29] First M. B. (2013). Diagnostic and statistical manual of mental disorders, 5th edition, and clinical utility. J. Nerv. Ment. Dis. 201, 727–729. doi: 10.1097/NMD.0b013e3182a2168a, PMID: 23995026

[ref30] Galluzzi L. Baehrecke E. H. Ballabio A. Boya P. Bravo-San Pedro J. M. Cecconi F.. (2017). Molecular definitions of autophagy and related processes. EMBO J. 36, 1811–1836. doi: 10.15252/embj.201796697, PMID: 28596378 PMC5494474

[ref31] Gardner A. Boles R. G. (2011). Beyond the serotonin hypothesis: mitochondria, inflammation and neurodegeneration in major depression and affective spectrum disorders. Prog. Neuro-Psychopharmacol. Biol. Psychiatry 35, 730–743. doi: 10.1016/j.pnpbp.2010.07.030 20691744

[ref32] Gatliff J. East D. Crosby J. Abeti R. Harvey R. Craigen W.. (2014). TSPO interacts with VDAC1 and triggers a ROS-mediated inhibition of mitochondrial quality control. Autophagy 10, 2279–2296. doi: 10.4161/15548627.2014.991665 25470454 PMC4502750

[ref33] Gong Y. Chai Y. Ding J. H. Sun X. L. Hu G. (2011). Chronic mild stress damages mitochondrial ultrastructure and function in mouse brain. Neurosci. Lett. 488, 76–80. doi: 10.1016/j.neulet.2010.11.006, PMID: 21070835

[ref34] Grimm A. Eckert A. (2017). Brain aging and neurodegeneration: from a mitochondrial point of view. J. Neurochem. 143, 418–431. doi: 10.1111/jnc.14037, PMID: 28397282 PMC5724505

[ref35] Guo L. Jiang Z. M. Sun R. X. Pang W. Zhou X. Du M. L.. (2022). Repeated social defeat stress inhibits development of hippocampus neurons through mitophagy and autophagy. Brain Res. Bull. 182, 111–117. doi: 10.1016/j.brainresbull.2022.01.009, PMID: 35114337

[ref36] Han X. Xu T. Fang Q. Zhang H. Yue L. Hu G.. (2021). Quercetin hinders microglial activation to alleviate neurotoxicity via the interplay between NLRP3 inflammasome and mitophagy. Redox Biol. 44:102010. doi: 10.1016/j.redox.2021.102010, PMID: 34082381 PMC8182123

[ref37] Hao L. Haitao W. Aijun X. Dong C. Jigang L. Quan K. (2013). Changes and mechanisms of autophagy in hippocampal neurons of depression model rats%. J Jilin Univ. (Med. Ed). 39, 672–675. Available at: https://kns.cnki.net/kcms/detail/22.1342.R.20130702.1407.002.html

[ref38] Harper J. W. Ordureau A. Heo J. M. (2018). Building and decoding ubiquitin chains for mitophagy. Nat. Rev. Mol. Cell Biol. 19, 93–108. doi: 10.1038/nrm.2017.129, PMID: 29358684

[ref39] Hertz L. Peng L. Dienel G. A. (2007). Energy metabolism in astrocytes: high rate of oxidative metabolism and spatiotemporal dependence on glycolysis/glycogenolysis. J. Cereb. Blood Flow Metab. 27, 219–249. doi: 10.1038/sj.jcbfm.9600343, PMID: 16835632

[ref40] Hetrick S. E. McKenzie J. E. Bailey A. P. Sharma V. Moller C. I. Badcock P. B.. (2021). New generation antidepressants for depression in children and adolescents: a network meta-analysis. Cochrane Database Syst. Rev. 5:CD013674. doi: 10.1002/14651858.CD013674.pub2, PMID: 34029378 PMC8143444

[ref41] Hollis F. Pope B. S. Gorman-Sandler E. Wood S. K. (2022). Neuroinflammation and mitochondrial dysfunction link social stress to depression. Curr. Top. Behav. Neurosci. 54, 59–93. doi: 10.1007/7854_2021_300 35184261

[ref42] Hollville E. Carroll R. G. Cullen S. P. Martin S. J. (2014). Bcl-2 family proteins participate in mitochondrial quality control by regulating Parkin/PINK1-dependent mitophagy. Mol. Cell 55, 451–466. doi: 10.1016/j.molcel.2014.06.001, PMID: 24999239

[ref43] Jernigan C. S. Goswami D. B. Austin M. C. Iyo A. H. Chandran A. Stockmeier C. A.. (2011). The mTOR signaling pathway in the prefrontal cortex is compromised in major depressive disorder. Prog. Neuro-Psychopharmacol. Biol. Psychiatry 35, 1774–1779. doi: 10.1016/j.pnpbp.2011.05.010 PMC3154612 21635931

[ref44] Jin S. M. Lazarou M. Wang C. Kane L. A. Narendra D. P. Youle R. J. (2010). Mitochondrial membrane potential regulates PINK1 import and proteolytic destabilization by PARL. J. Cell Biol. 191, 933–942. doi: 10.1083/jcb.201008084, PMID: 21115803 PMC2995166

[ref45] Jin X. Zhu L. Lu S. Li C. Bai M. Xu E.. (2023). Baicalin ameliorates CUMS-induced depression-like behaviors through activating AMPK/PGC-1alpha pathway and enhancing NIX-mediated mitophagy in mice. Eur. J. Pharmacol. 938:175435. doi: 10.1016/j.ejphar.2022.175435, PMID: 36463946

[ref46] Kane L. A. Lazarou M. Fogel A. I. Li Y. Yamano K. Sarraf S. A.. (2014). PINK1 phosphorylates ubiquitin to activate Parkin E3 ubiquitin ligase activity. J. Cell Biol. 205, 143–153. doi: 10.1083/jcb.201402104, PMID: 24751536 PMC4003245

[ref47] Kann O. Kovacs R. (2007). Mitochondria and neuronal activity. Am. J. Physiol. Cell Physiol. 292, C641–C657. doi: 10.1152/ajpcell.00222.2006, PMID: 17092996

[ref48] Karabatsiakis A. Bock C. Salinas-Manrique J. Kolassa S. Calzia E. Dietrich D. E.. (2014). Mitochondrial respiration in peripheral blood mononuclear cells correlates with depressive subsymptoms and severity of major depression. Transl. Psychiatry 4:e397. doi: 10.1038/tp.2014.44, PMID: 26126180 PMC4080325

[ref49] Keller J. Gomez R. Williams G. Lembke A. Lazzeroni L. Murphy G. M. Jr.. (2017). HPA axis in major depression: cortisol, clinical symptomatology and genetic variation predict cognition. Mol. Psychiatry 22, 527–536. doi: 10.1038/mp.2016.120, PMID: 27528460 PMC5313380

[ref50] Kerr J. S. Adriaanse B. A. Greig N. H. Mattson M. P. Cader M. Z. Bohr V. A.. (2017). Mitophagy and Alzheimer's Disease: cellular and molecular mechanisms. Trends Neurosci. 40, 151–166. doi: 10.1016/j.tins.2017.01.002, PMID: 28190529 PMC5341618

[ref51] Khan M. Baussan Y. Hebert-Chatelain E. (2023). Connecting dots between mitochondrial dysfunction and depression. Biomol. Ther. 13:695. doi: 10.3390/biom13040695, PMID: 37189442 PMC10135685

[ref52] Kim Y. K. (2016). Molecular neurobiology of major depressive disorder. Prog. Neuro-Psychopharmacol. Biol. Psychiatry 64, 275–276. doi: 10.1016/j.pnpbp.2015.07.004 26169576

[ref53] Kong H. Zeng X. N. Fan Y. Yuan S. T. Ge S. Xie W. P.. (2014). Aquaporin-4 knockout exacerbates corticosterone-induced depression by inhibiting astrocyte function and hippocampal neurogenesis. CNS Neurosci. Ther. 20, 391–402. doi: 10.1111/cns.12222, PMID: 24422972 PMC6493035

[ref54] Kwon Y. Bang Y. Moon S. H. Kim A. Choi H. J. (2020). Amitriptyline interferes with autophagy-mediated clearance of protein aggregates via inhibiting autophagosome maturation in neuronal cells. Cell Death Dis. 11:874. doi: 10.1038/s41419-020-03085-6 33070168 PMC7568721

[ref55] Lee Y. Lee H. Y. Hanna R. A. Gustafsson A. B. (2011). Mitochondrial autophagy by Bnip 3 involves Drp 1-mediated mitochondrial fission and recruitment of Parkin in cardiac myocytes. Am. J. Physiol. Heart Circ. Physiol. 301, H1924–H1931. doi: 10.1152/ajpheart.00368.2011, PMID: 21890690 PMC3213962

[ref56] Lemasters J. J. (2005). Selective mitochondrial autophagy, or mitophagy, as a targeted defense against oxidative stress, mitochondrial dysfunction, and aging. Rejuvenation Res. 8, 3–5. doi: 10.1089/rej.2005.8.3, PMID: 15798367

[ref57] Li Y. C. Wang L. L. Pei Y. Y. Shen J. D. Li H. B. Wang B. Y.. (2015). Baicalin decreases SGK1 expression in the hippocampus and reverses depressive-like behaviors induced by corticosterone. Neuroscience 311, 130–137. doi: 10.1016/j.neuroscience.2015.10.023, PMID: 26480816

[ref58] Li K. Yan L. Zhang Y. Yang Z. Zhang C. Li Y.. (2020). Seahorse treatment improves depression-like behavior in mice exposed to CUMS through reducing inflammation/oxidants and restoring neurotransmitter and neurotrophin function. J. Ethnopharmacol. 250:112487. doi: 10.1016/j.jep.2019.112487, PMID: 31857128

[ref59] Li D. Zheng J. Wang M. Feng L. Liu Y. Yang N.. (2016). Wuling powder prevents the depression-like behavior in learned helplessness mice model through improving the TSPO mediated-mitophagy. J. Ethnopharmacol. 186, 181–188. doi: 10.1016/j.jep.2016.03.065, PMID: 27063986

[ref60] Li C. Zhu Y. Wu Y. Fu M. Wu Y. Wu Y.. (2021). Oridonin alleviates LPS-induced depression by inhibiting NLRP3 Inflammasome via activation of autophagy. Front Med (Lausanne). 8:813047. doi: 10.3389/fmed.2021.813047, PMID: 35096901 PMC8790066

[ref61] Lin Q. Li S. Jiang N. Shao X. Zhang M. Jin H.. (2019). PINK1-parkin pathway of mitophagy protects against contrast-induced acute kidney injury via decreasing mitochondrial ROS and NLRP3 inflammasome activation. Redox Biol. 26:101254. doi: 10.1016/j.redox.2019.101254, PMID: 31229841 PMC6597739

[ref62] Liu P. Du J. (2020). Oridonin is an antidepressant molecule working through the PPAR-gamma/AMPA receptor signaling pathway. Biochem. Pharmacol. 180:114136. doi: 10.1016/j.bcp.2020.114136, PMID: 32628930

[ref63] Liu L. Feng D. Chen G. Chen M. Zheng Q. Song P.. (2012). Mitochondrial outer-membrane protein FUNDC1 mediates hypoxia-induced mitophagy in mammalian cells. Nat. Cell Biol. 14, 177–185. doi: 10.1038/ncb2422, PMID: 22267086

[ref64] Liu J. Liu L. Han Y. S. Yi J. Guo C. Zhao H. Q.. (2021). The molecular mechanism underlying mitophagy-mediated hippocampal neuron apoptosis in diabetes-related depression. J. Cell. Mol. Med. 25, 7342–7353. doi: 10.1111/jcmm.16763, PMID: 34213839 PMC8335699

[ref65] Lizama B. N. Chu C. T. (2021). Neuronal autophagy and mitophagy in Parkinson's disease. Mol. Asp. Med. 82:100972. doi: 10.1016/j.mam.2021.100972, PMID: 34130867 PMC8665948

[ref66] Lu J. J. Wu P. F. He J. G. Li Y. K. Long L. H. Yao X. P.. (2023). BNIP3L/NIX-mediated mitophagy alleviates passive stress-coping behaviors induced by tumor necrosis factor-alpha. Mol. Psychiatry. doi: 10.1038/s41380-023-02008-z, PMID: 36914810

[ref67] Lv M. Wang C. Li F. Peng J. Wen B. Gong Q.. (2017). Structural insights into the recognition of phosphorylated FUNDC1 by LC3B in mitophagy. Protein Cell 8, 25–38. doi: 10.1007/s13238-016-0328-8, PMID: 27757847 PMC5233613

[ref68] Lyu D. Wang F. Zhang M. Yang W. Huang H. Huang Q.. (2022). Ketamine induces rapid antidepressant effects via the autophagy-NLRP3 inflammasome pathway. Psychopharmacology 239, 3201–3212. doi: 10.1007/s00213-022-06201-w, PMID: 35925279

[ref69] Ma Z. Chen C. Tang P. Zhang H. Yue J. Yu Z. (2017). BNIP3 induces apoptosis and protective autophagy under hypoxia in esophageal squamous cell carcinoma cell lines: BNIP3 regulates cell death. Dis. Esophagus 30, 1–8. doi: 10.1093/dote/dox059 28859361

[ref70] Malhi G. S. Mann J. J. (2018). Depression. Lancet 392, 2299–2312. doi: 10.1016/S0140-6736(18)31948-2, PMID: 30396512

[ref71] Malpartida A. B. Williamson M. Narendra D. P. Wade-Martins R. Ryan B. J. (2021). Mitochondrial dysfunction and Mitophagy in Parkinson's Disease: from mechanism to therapy. Trends Biochem. Sci. 46, 329–343. doi: 10.1016/j.tibs.2020.11.007, PMID: 33323315

[ref72] Mancuso M. Orsucci D. Ienco E. C. Pini E. Choub A. Siciliano G. (2013). Psychiatric involvement in adult patients with mitochondrial disease. Neurol. Sci. 34, 71–74. doi: 10.1007/s10072-011-0901-0, PMID: 22193419

[ref73] Marwaha S. Palmer E. Suppes T. Cons E. Young A. H. Upthegrove R. (2023). Novel and emerging treatments for major depression. Lancet 401, 141–153. doi: 10.1016/S0140-6736(22)02080-3, PMID: 36535295

[ref74] Marx W. Lane M. Hockey M. Aslam H. Berk M. Walder K.. (2021). Diet and depression: exploring the biological mechanisms of action. Mol. Psychiatry 26, 134–150. doi: 10.1038/s41380-020-00925-x, PMID: 33144709

[ref75] Matsushima M. Fujiwara T. Takahashi E. Minaguchi T. Eguchi Y. Tsujimoto Y.. (1998). Isolation, mapping, and functional analysis of a novel human cDNA (BNIP3L) encoding a protein homologous to human NIP3. Genes Chromosomes Cancer 21, 230–235. doi: 10.1002/(SICI)1098-2264(199803)21:3<230::AID-GCC7>3.0.CO;2-0, PMID: 9523198

[ref76] McCarron R. M. Shapiro B. Rawles J. Luo J. (2021). Depression. Ann. Intern. Med. 174, ITC65–ITC80. doi: 10.7326/AITC202105180 33971098

[ref77] Meissner C. Lorenz H. Weihofen A. Selkoe D. J. Lemberg M. K. (2011). The mitochondrial intramembrane protease PARL cleaves human pink 1 to regulate pink 1 trafficking. J. Neurochem. 117, 856–867. doi: 10.1111/j.1471-4159.2011.07253.x, PMID: 21426348

[ref78] Meng W. (2020). Mechanism of Xiao Jian Zhong Tang inhibiting NLRP3 inflammatory body activation in the treatment of depression based on mitochondrial autophagy. doi: 10.26988/d.cnki.gcdzu.2020.000070

[ref79] Micheli L. Ceccarelli M. D'Andrea G. Tirone F. (2018). Depression and adult neurogenesis: positive effects of the antidepressant fluoxetine and of physical exercise. Brain Res. Bull. 143, 181–193. doi: 10.1016/j.brainresbull.2018.09.002, PMID: 30236533

[ref80] Morava E. Gardeitchik T. Kozicz T. de Boer L. Koene S. de Vries M. C.. (2010). Depressive behaviour in children diagnosed with a mitochondrial disorder. Mitochondrion 10, 528–533. doi: 10.1016/j.mito.2010.05.011, PMID: 20573558

[ref81] Ning B. Wang Z. Wu Q. Deng Q. Yang Q. Gao J.. (2023). Acupuncture inhibits autophagy and repairs synapses by activating the mTOR pathway in Parkinson's disease depression model rats. Brain Res. 1808:148320. doi: 10.1016/j.brainres.2023.148320, PMID: 36914042

[ref82] Novak I. Kirkin V. McEwan D. G. Zhang J. Wild P. Rozenknop A.. (2010). Nix is a selective autophagy receptor for mitochondrial clearance. EMBO Rep. 11, 45–51. doi: 10.1038/embor.2009.256, PMID: 20010802 PMC2816619

[ref83] Palikaras K. Lionaki E. Tavernarakis N. (2018). Mechanisms of mitophagy in cellular homeostasis, physiology and pathology. Nat. Cell Biol. 20, 1013–1022. doi: 10.1038/s41556-018-0176-2, PMID: 30154567

[ref84] Parsons M. J. Green D. R. (2010). Mitochondria in cell death. Essays Biochem. 47, 99–114. doi: 10.1042/bse0470099, PMID: 20533903

[ref85] Pekny M. Pekna M. Messing A. Steinhauser C. Lee J. M. Parpura V.. (2016). Astrocytes: a central element in neurological diseases. Acta Neuropathol. 131, 323–345. doi: 10.1007/s00401-015-1513-1, PMID: 26671410

[ref86] Perez-Caballero L. Torres-Sanchez S. Bravo L. Mico J. A. Berrocoso E. (2014). Fluoxetine: a case history of its discovery and preclinical development. Expert Opin Drug Discov. 9, 567–578. doi: 10.1517/17460441.2014.907790, PMID: 24738878

[ref87] Pickles S. Vigie P. Youle R. J. (2018). Mitophagy and quality control mechanisms in mitochondrial maintenance. Curr. Biol. 28, R170–R185. doi: 10.1016/j.cub.2018.01.004, PMID: 29462587 PMC7255410

[ref88] Pickrell A. M. Youle R. J. (2015). The roles of PINK1, parkin, and mitochondrial fidelity in Parkinson's disease. Neuron 85, 257–273. doi: 10.1016/j.neuron.2014.12.007, PMID: 25611507 PMC4764997

[ref89] Pinna G. Rasmusson A. M. (2012). Up-regulation of neurosteroid biosynthesis as a pharmacological strategy to improve behavioural deficits in a putative mouse model of post-traumatic stress disorder. J. Neuroendocrinol. 24, 102–116. doi: 10.1111/j.1365-2826.2011.02234.x, PMID: 21981145 PMC3245370

[ref90] Quiroz J. A. Gray N. A. Kato T. Manji H. K. (2008). Mitochondrially mediated plasticity in the pathophysiology and treatment of bipolar disorder. Neuropsychopharmacology 33, 2551–2565. doi: 10.1038/sj.npp.1301671, PMID: 18235426

[ref91] Rangaraju V. Calloway N. Ryan T. A. (2014). Activity-driven local ATP synthesis is required for synaptic function. Cells 156, 825–835. doi: 10.1016/j.cell.2013.12.042, PMID: 24529383 PMC3955179

[ref92] Ranjan K. Pathak C. (2016). Expression of cFLIPL determines the basal interaction of Bcl-2 with Beclin-1 and regulates p 53 dependent ubiquitination of Beclin-1 during Autophagic stress. J. Cell. Biochem. 117, 1757–1768. doi: 10.1002/jcb.25474, PMID: 26682748

[ref93] Reddy A. P. Sawant N. Morton H. Kshirsagar S. Bunquin L. E. Yin X.. (2021a). Selective serotonin reuptake inhibitor citalopram ameliorates cognitive decline and protects against amyloid beta-induced mitochondrial dynamics, biogenesis, autophagy, mitophagy and synaptic toxicities in a mouse model of Alzheimer's disease. Hum. Mol. Genet. 30, 789–810. doi: 10.1093/hmg/ddab091, PMID: 33791799 PMC8161521

[ref94] Reddy A. P. Yin X. Sawant N. Reddy P. H. (2021b). Protective effects of antidepressant citalopram against abnormal APP processing and amyloid beta-induced mitochondrial dynamics, biogenesis, mitophagy and synaptic toxicities in Alzheimer's disease. Hum. Mol. Genet. 30, 847–864. doi: 10.1093/hmg/ddab054 33615359 PMC8355469

[ref95] Rezin G. T. Goncalves C. L. Daufenbach J. F. Fraga D. B. Santos P. M. Ferreira G. K.. (2009). Acute administration of ketamine reverses the inhibition of mitochondrial respiratory chain induced by chronic mild stress. Brain Res. Bull. 79, 418–421. doi: 10.1016/j.brainresbull.2009.03.010, PMID: 19393724

[ref96] Rush A. J. Warden D. Wisniewski S. R. Fava M. Trivedi M. H. Gaynes B. N.. (2009). STAR*D: revising conventional wisdom. CNS Drugs 23, 627–647. doi: 10.2165/00023210-200923080-00001 19594193

[ref97] Ryan K. M. Doody E. McLoughlin D. M. (2023). Whole blood mitochondrial DNA copy number in depression and response to electroconvulsive therapy. Prog. Neuro-Psychopharmacol. Biol. Psychiatry 121:110656. doi: 10.1016/j.pnpbp.2022.110656 36216200

[ref98] Scaini G. Mason B. L. Diaz A. P. Jha M. K. Soares J. C. Trivedi M. H.. (2022). Dysregulation of mitochondrial dynamics, mitophagy and apoptosis in major depressive disorder: does inflammation play a role? Mol. Psychiatry 27, 1095–1102. doi: 10.1038/s41380-021-01312-w, PMID: 34650203

[ref99] Schweers R. L. Zhang J. Randall M. S. Loyd M. R. Li W. Dorsey F. C.. (2007). NIX is required for programmed mitochondrial clearance during reticulocyte maturation. Proc. Natl. Acad. Sci. U. S. A. 104, 19500–19505. doi: 10.1073/pnas.0708818104, PMID: 18048346 PMC2148318

[ref100] Setiawan E. Wilson A. A. Mizrahi R. Rusjan P. M. Miler L. Rajkowska G.. (2015). Role of translocator protein density, a marker of neuroinflammation, in the brain during major depressive episodes. JAMA Psychiatry 72, 268–275. doi: 10.1001/jamapsychiatry.2014.2427, PMID: 25629589 PMC4836849

[ref101] Shen F. Song Z. Xie P. Li L. Wang B. Peng D.. (2021). Polygonatum sibiricum polysaccharide prevents depression-like behaviors by reducing oxidative stress, inflammation, and cellular and synaptic damage. J. Ethnopharmacol. 275:114164. doi: 10.1016/j.jep.2021.114164, PMID: 33932516

[ref102] Shu X. Sun Y. Sun X. Zhou Y. Bian Y. Shu Z.. (2019). The effect of fluoxetine on astrocyte autophagy flux and injured mitochondria clearance in a mouse model of depression. Cell Death Dis. 10:577. doi: 10.1038/s41419-019-1813-9 31371719 PMC6675792

[ref103] Song W. M. Colonna M. (2018). The identity and function of microglia in neurodegeneration. Nat. Immunol. 19, 1048–1058. doi: 10.1038/s41590-018-0212-1, PMID: 30250185

[ref104] Spinelli J. B. Haigis M. C. (2018). The multifaceted contributions of mitochondria to cellular metabolism. Nat. Cell Biol. 20, 745–754. doi: 10.1038/s41556-018-0124-1, PMID: 29950572 PMC6541229

[ref105] Sprague A. H. Khalil R. A. (2009). Inflammatory cytokines in vascular dysfunction and vascular disease. Biochem. Pharmacol. 78, 539–552. doi: 10.1016/j.bcp.2009.04.029, PMID: 19413999 PMC2730638

[ref106] Su W.-J. Zhang Y. Chen Y. Gong H. Lian Y.-J. Peng W.. (2017). NLRP3 gene knockout blocks NF-κB and MAPK signaling pathway in CUMS-induced depression mouse model. Behav. Brain Res. 322, 1–8. doi: 10.1016/j.bbr.2017.01.018, PMID: 28093255

[ref107] Suzuki K. Nakamura K. Shimizu Y. Yokoi Y. Ohira S. Hagiwara M.. (2021). Decrease of alpha-defensin impairs intestinal metabolite homeostasis via dysbiosis in mouse chronic social defeat stress model. Sci. Rep. 11:9915. doi: 10.1038/s41598-021-89308-y 33972646 PMC8110768

[ref108] Taene A. Khalili-Tanha G. Esmaeili A. Mobasheri L. Kooshkaki O. Jafari S.. (2020). The Association of Major Depressive Disorder with activation of NLRP3 Inflammasome, lipid peroxidation, and Total antioxidant capacity. J. Mol. Neurosci. 70, 65–70. doi: 10.1007/s12031-019-01401-0, PMID: 31515707

[ref109] The Lancet (2019). Icd-11. Lancet 393:2275. doi: 10.1016/S0140-6736(19)31205-X 31180012

[ref110] Thomas R. L. Kubli D. A. Gustafsson A. B. (2011). Bnip 3-mediated defects in oxidative phosphorylation promote mitophagy. Autophagy 7, 775–777. doi: 10.4161/auto.7.7.15536 21460627

[ref111] Todorova V. Blokland A. (2017). Mitochondria and synaptic plasticity in the mature and aging nervous system. Curr. Neuropharmacol. 15, 166–173. doi: 10.2174/1570159X14666160414111821, PMID: 27075203 PMC5327446

[ref112] Tripathi A. Scaini G. Barichello T. Quevedo J. Pillai A. (2021). Mitophagy in depression: pathophysiology and treatment targets. Mitochondrion 61, 1–10. doi: 10.1016/j.mito.2021.08.016, PMID: 34478906 PMC8962570

[ref113] Ulrich S. Ricken R. Buspavanich P. Schlattmann P. Adli M. (2020). Efficacy and adverse effects of tranylcypromine and tricyclic antidepressants in the treatment of depression: a systematic review and comprehensive Meta-analysis. J. Clin. Psychopharmacol. 40, 63–74. doi: 10.1097/JCP.0000000000001153, PMID: 31834088

[ref114] von Bernhardi R. Eugenin-von Bernhardi J. Flores B. Eugenin Leon J. (2016). Glial cells and integrity of the nervous system. Adv. Exp. Med. Biol. 949, 1–24. doi: 10.1007/978-3-319-40764-7_1 27714682

[ref115] Wander S. A. Hennessy B. T. Slingerland J. M. (2011). Next-generation mTOR inhibitors in clinical oncology: how pathway complexity informs therapeutic strategy. J. Clin. Invest. 121, 1231–1241. doi: 10.1172/JCI44145, PMID: 21490404 PMC3069769

[ref116] Wang G. Liu Y. Zhu X. Lin K. Li M. Wu Z.. (2022). Knockdown of mi RNA-134-5p rescues dendritic deficits by promoting AMPK-mediated mitophagy in a mouse model of depression. Neuropharmacology 214:109154. doi: 10.1016/j.neuropharm.2022.109154, PMID: 35659969

[ref117] Wang B. Shi H. Yang B. Miao Z. Sun M. Yang H.. (2023). The mitochondrial ahi 1/GR participates the regulation on mt DNA copy numbers and brain ATP levels and modulates depressive behaviors in mice. Cell Commun. Signal 21:21. doi: 10.1186/s12964-022-01034-8 36691038 PMC9869592

[ref118] Wei Y. Pattingre S. Sinha S. Bassik M. Levine B. (2008). JNK1-mediated phosphorylation of Bcl-2 regulates starvation-induced autophagy. Mol. Cell 30, 678–688. doi: 10.1016/j.molcel.2008.06.001, PMID: 18570871 PMC2478643

[ref119] Wei Q. Zhou W. Zheng J. Li D. Wang M. Feng L.. (2020). Antidepressant effects of 3-(3, 4-methylenedioxy-5-trifluoromethyl phenyl)-2E-propenoic acid isobutyl amide involve TSPO-mediated mitophagy signalling pathway. Basic Clin. Pharmacol. Toxicol. 127, 380–388. doi: 10.1111/bcpt.13452, PMID: 32511877

[ref120] Weixing K. (2019). Changes in Mitochondrial Autophagy and Inflammasome Related Genes in Patients with Depression Before and After Electroconvulsive Therapy.

[ref121] Winden K. D. Ebrahimi-Fakhari D. Sahin M. (2018). Abnormal mTOR activation in autism. Annu. Rev. Neurosci. 41, 1–23. doi: 10.1146/annurev-neuro-080317-061747, PMID: 29490194

[ref122] Wu M. Zhao L. Wang Y. Guo Q. An Q. Geng J.. (2022). Ketamine regulates the autophagy flux and polarization of microglia through the HMGB1-RAGE Axis and exerts antidepressant effects in mice. J. Neuropathol. Exp. Neurol. 81, 931–942. doi: 10.1093/jnen/nlac035, PMID: 35582883

[ref123] Xu Y. Wang Z. You W. Zhang X. Li S. Barish P. A.. (2010). Antidepressant-like effect of trans-resveratrol: involvement of serotonin and noradrenaline system. Eur. Neuropsychopharmacol. 20, 405–413. doi: 10.1016/j.euroneuro.2010.02.013, PMID: 20353885

[ref124] Xu L. Z. Xu D. F. Han Y. Liu L. J. Sun C. Y. Deng J. H.. (2017). BDNF-GSK-3beta-beta-catenin pathway in the mPFC is involved in antidepressant-like effects of Morinda officinalis oligosaccharides in rats. Int. J. Neuropsychopharmacol. 20, 83–93. doi: 10.1093/ijnp/pyw088, PMID: 27729466 PMC5737867

[ref125] Yamano K. Matsuda N. Tanaka K. (2016). The ubiquitin signal and autophagy: an orchestrated dance leading to mitochondrial degradation. EMBO Rep. 17, 300–316. doi: 10.15252/embr.201541486, PMID: 26882551 PMC4772979

[ref126] Yamano K. Youle R. J. (2013). PINK1 is degraded through the N-end rule pathway. Autophagy 9, 1758–1769. doi: 10.4161/auto.24633 24121706 PMC4028335

[ref127] Yang L. Ao Y. Li Y. Dai B. Li J. Duan W.. (2023). Morinda officinalis oligosaccharides mitigate depression-like behaviors in hypertension rats by regulating Mfn 2-mediated mitophagy. J. Neuroinflammation 20:31. doi: 10.1186/s12974-023-02715-y 36765376 PMC9912533

[ref128] Yang M. He Y. Deng S. Xiao L. Tian M. Xin Y.. (2021). Mitochondrial quality control: a pathophysiological mechanism and therapeutic target for stroke. Front. Mol. Neurosci. 14:786099. doi: 10.3389/fnmol.2021.786099, PMID: 35153669 PMC8832032

[ref129] Yuan Q. Li Y. Deng X. Shi H. Zhao Z. Wang C.. (2019). Effects of Xingpi Kaiyu Fang on ATP, Na/K-ATPase, and respiratory chain complexes of Hippocampus and gastrocnemius muscle in depressed rats. Evid. Based Complement. Alternat. Med. 2019:6054926. doi: 10.1155/2019/6054926, PMID: 30719062 PMC6335795

[ref130] Zhang Z. Cai X. Yao Z. Wen F. Fu Z. Zhang J.. (2020). EA ameliorated depressive behaviors in CUMS rats and was related to its suppressing autophagy in the Hippocampus. Neural Plast. 2020:8860968. doi: 10.1155/2020/8860968, PMID: 33029121 PMC7527933

[ref131] Zhang J. H. Xin H. L. Xu Y. M. Shen Y. He Y. Q. Hsien Y.. (2018). Morinda officinalis how – a comprehensive review of traditional uses, phytochemistry and pharmacology. J. Ethnopharmacol. 213, 230–255. doi: 10.1016/j.jep.2017.10.028, PMID: 29126988

[ref132] Zhang G. Xu S. Zhang Z. Zhang Y. Wu Y. An J.. (2020). Identification of key genes and the pathophysiology associated with major depressive disorder patients based on integrated bioinformatics analysis. Front. Psych. 11:192. doi: 10.3389/fpsyt.2020.00192 PMC7146847 32317989

[ref133] Zorov D. B. Juhaszova M. Sollott S. J. (2014). Mitochondrial reactive oxygen species (ROS) and ROS-induced ROS release. Physiol. Rev. 94, 909–950. doi: 10.1152/physrev.00026.2013, PMID: 24987008 PMC4101632

[ref134] Zschocke J. Zimmermann N. Berning B. Ganal V. Holsboer F. Rein T. (2011). Antidepressant drugs diversely affect autophagy pathways in astrocytes and neurons--dissociation from cholesterol homeostasis. Neuropsychopharmacology 36, 1754–1768. doi: 10.1038/npp.2011.57, PMID: 21508931 PMC3138654

